# Personal Care Aide Staffing in U.S. Residential Care Communities

**DOI:** 10.1093/geroni/igaf122.4244

**Published:** 2025-12-31

**Authors:** Erh-Chi Hsu, Katherine Kennedy, David Mohr

**Affiliations:** Johns Hopkins University, Baltimore, Maryland, United States; Providence VA Healthcare System, Providence, Rhode Island, United States; Veterans Health Administration, Mason, Ohio, United States

## Abstract

Residential care communities (RCCs) employ approximately 660,000 personal care aides—nearly three-quarters of the direct care workforce in these settings—yet these workers receive minimal training and are not licensed healthcare professionals. Despite their essential role, national patterns of aide staffing and their relationship to RCC characteristics remain unknown. This study examines how training requirements and organizational factors are associated with aide hours per resident day (HPRD) using data from the 2022 National Post-acute and Long-term Care Study. A weighted sample of 518 RCCs was analyzed using regression models. Higher aide staffing levels were significantly associated with RCCs that reimbursed aides for initial training (β = 0.84, SE = 0.36) and with facilities operating at greater than 85% occupancy (β = 0.82, SE = 0.34). In contrast, lower staffing levels were observed in RCCs requiring more than 60 hours of initial training (β=-1.36, SE = 0.58), serving 1–25% (β=-1.19, SE = 0.57) or ≥ 25% (β=-2.01, SE = 0.51) Medicaid-supported residents, and those with more than 50 beds (β=-2.14, SE = 0.30). Neither continuing education requirements nor ownership type were significantly associated with staffing. Findings underscore the importance of supporting aide training reimbursement in association with higher staffing ratios. However, more extensive initial training requirements may reflect an expectation of greater responsibility per aide, potentially reducing staffing needs. Further research is needed to explore the trade-offs between training hours and staffing levels and to better understand the influence of Medicaid participation on workforce capacity in RCCs.

